# Relapsing Demyelinating Syndromes in Children: A Practical Review of Neuroradiological Mimics

**DOI:** 10.3389/fneur.2020.00627

**Published:** 2020-08-04

**Authors:** Sahil Chhabda, Prateek Malik, Nihaal Reddy, Karthik Muthusamy, David Mirsky, Sniya Sudhakar, Kshitij Mankad

**Affiliations:** ^1^Department of Radiology, Great Ormond Street Hospital, London, United Kingdom; ^2^Christian Medical College & Hospital, Vellore, India; ^3^Rainbow Children's Hospital, Hyderabad, India; ^4^Children's Hospital Colorado, Aurora, CO, United States; ^5^Associate Honorary Professor, Institute of Child Health, University College London, London, United Kingdom

**Keywords:** demyelimating disease, pediatric, multiple scleorsis, ADEM, MS, MOG, AQP4, mimics

## Abstract

Relapsing demyelinating syndromes (RDS) in children encompass a diverse spectrum of entities including multiple sclerosis (MS) acute disseminated encephalomyelitis (ADEM), aquaporin-4 antibody associated neuromyelitis optica spectrum disorder (AQP4-NMOSD) and myelin oligodendrocyte glycoprotein antibody disease (MOG-AD). In addition to these, there are “antibody-negative” demyelinating syndromes which are yet to be fully characterized and defined. The paucity of specific biomarkers and overlap in clinical presentations makes the distinction between these disease entities difficult at initial presentation and, as such, there is a heavy reliance on magnetic resonance imaging (MRI) findings to satisfy the criteria for treatment initiation and optimization. Misdiagnosis is not uncommon and is usually related to the inaccurate application of criteria or failure to identify potential clinical and radiological mimics. It is also notable that there are instances where AQP4 and MOG antibody testing may be falsely negative during initial clinical episodes, further complicating the issue. This article illustrates the typical clinico-radiological phenotypes associated with the known pediatric RDS at presentation and describes the neuroimaging mimics of these using a pattern-based approach in the brain, optic nerves, and spinal cord. Practical guidance on key distinguishing features in the form of clinical and radiological red flags are incorporated. A subsection on clinical mimics with characteristic imaging patterns that assist in establishing alternative diagnoses is also included.

## What are the Typical Radiological Features of Relapsing Inflammatory Demyelinating Disorders in Children?

### Multiple Sclerosis (MS)

MS is the most common RDS in children. The diagnosis of MS is based on the revised 2017 McDonald criteria which integrates clinical, radiological, and laboratory findings (1). The McDonald criteria perform well in identifying pediatric patients with MS (2), however they are not validated in patients under 11 years (1). Additionally, these criteria should only be applied when alternative causes have been excluded by clinical assessment and laboratory testing.

The radiological appearances of pediatric MS are largely similar to those observed in adult cohorts (3). Some unique imaging findings have however been described in pediatric MS, such as a higher lesion burden at presentation when compared to adults, particularly involving the brainstem and cerebellum. In prepubertal children demyelinating lesions are usually larger, confluent, have ill-defined borders and show a higher predilection for deep gray matter structures. Tumefactive (>2 cm) lesions are also more common in children (4–8).

In typical cases, MS lesions are small, well-defined, round or ovoid in shape and located in the periventricular white matter, juxta/intracortical regions, brainstem, and cerebellum, and/or in the spinal cord (9). The periventricular lesions abut the lateral ventricular margin with no normal white matter interspersed in between. They are orientated perpendicular to the ventricular margin along the deep medullary veins and have been termed “Dawson's fingers.” Likewise, the juxta/intracortical lesions should abut the cortex or be present within the cortex.

Contrast enhancement is common and variable and may be nodular, or demonstrate an open or closed ring-like morphology. Enhancement may persist for up to 2–8 weeks (9).

Spinal cord lesions are typically short segment (usually less than two vertebral heights), peripheral (or eccentric) on axial imaging, and cover less than half the cord circumference. A predilection for the cervical and thoracic cord has been noted (3).

Unlike in adults, optic nerve involvement in children, especially in those under 10 years of age tends to be more commonly bilateral with severe loss of visual acuity (10, 11). However, some studies dispute this (12). Bilateral involvement and white matter lesions on MRI at presentation, irrespective of the number, are associated with a significant risk of development of MS subsequently (11, 12). On MRI, there is T2 signal hyperintensity, with or without swelling or contrast enhancement. Optic nerve atrophy can be seen in the chronic phase.

### Aquaporin-4 Antibody Neuromyelitis Optica Spectrum Disorder (AQP4-NMOSD)

Aquaporin-4 (AQP4) is a membrane protein that assists with the transfer of water molecules across cell membranes. NMO-IgG targets the water channel AQP4 and is positive by serology in up to 70% of NMOSD patients. The diagnosis of AQP4-NMOSD is based on the 2015 international consensus criteria which comprises of core clinical characteristics, AQP4 antibody status, and MRI features (13). These criteria are applicable to both children and adults (14).

Specific to neuroimaging, the absence of juxtacortical/cortical lesions, absence of periventricular lesions, absence of Dawson's fingers, presence of longitudinally extensive transverse myelitis and presence of periependymal lesions along lateral ventricles supports the diagnosis of AQP4-NMOSD (15).

Other regions typically involved in the disease process, and on imaging are regions of high AQP4 expressivity and are located in the periependymal region surrounding the 3rd ventricle and cerebral aqueduct, dorsal brainstem adjacent to the 4th ventricle including the area postrema and nucleus tractus solitaries (9, 16).

The classically described findings are present in ~50% of cases. Other brain imaging patterns in AQP4-NMOSD include large hemispheric lesions, longitudinally extensive lesions along white matter tracts specifically corticospinal tracts, and, at times, even normal appearances.

Spinal cord involvement is usually in the form of longitudinally extensive transverse myelitis (LETM) involving more than three vertebral segments. The lesions often span >50% of the cross-section of the cord and demonstrate a central-predominant cord distribution. Short segment involvement has, however, also been described in a third of cases (15).

Optic nerve involvement is most commonly longitudinally extensive and bilateral, with a propensity for intracranial segments including the optic chiasm (17).

### Myelin Oligodendrocyte Glycoprotein Antibody Disease (MOG-AD)

MOG-AD represents a group of inflammatory demyelinating disorders united by the presence of IgG antibodies to myelin oligodendrocyte glycoprotein. MOG tends to affect younger children who presenting clinically with an ADEM-like picture. Older patients (>9 years) are more likely to present with optic neuritis or an AQP4-NMOSD-like picture (18).

The clinical presentations in MOG-AD are heterogeneous. Seizures have been described as a presenting clinical feature in MOG-AD with a higher frequency when compared to other RDS, namely AQP4-NMOSD and MS. Hypothesized theories for seizures associated with MOG-AD are cortical involvement by an encephalitic process, and also the co-existence of anti N-Methyl D-Aspartate antibodies (19).

The brain lesions on imaging are often large, ill-defined, and involve the white matter. There is variable deep gray matter involvement, with a predilection for the thalamus (18). Cortical involvement with or without meningeal enhancement has been described as a rare but distinct pattern in MOG-AD, and is characterized on imaging as FLAIR hyperintensity and swelling with reduced diffusivity (17, 20–22).

Spinal cord lesions are typically longitudinally extensive. Unlike other RDS, there is a predilection for the conus medullaris (23).

Optic neuritis with MOG-AD has distinct features, such as bilateral optic nerve involvement, anterior optic pathway predilection with optic disc swelling, and rapid visual impairment (24). Relapses with isolated optic neuritis are common.

**Figure 1:**
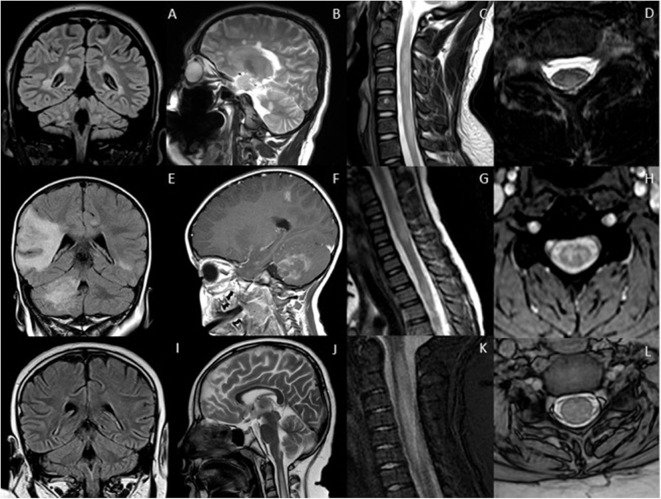
Typical appearances of RDS in children. MS—top row **(A–D)** Juxtacortical, periventricular and infratentorial brain lesions are readily appreciated on coronal T2-FLAIR **(A)** and sagittal T2-weighted images **(B)**. In the spine there are short segment eccentric lesions appreciated on sagittal and axial T2-weighted images (**C,D**, respectively). MOG-AD—middle row **(E–H)** Large confluent lesions with ill-defined enhancement are demonstrated within the brain on coronal T2-FLAIR **(E)** and contrast-enhanced sagittal T1-weighted images **(F)**. In the spine, there are lesions involving gray and white matter on sagittal and axial T2-weighted images (**G,H**, respectively). AQP4-NMOSD—bottom row **(I–L)** Brain lesions are present in areas of AQP4 expressivity. For example, in this case there is involvement of the area postrema on coronal T2-FLAIR **(I)** and sagittal T2-weighted images **(J)**. A lesion is also present in the upper spinal cord. In the spine there is longitudinally extensive transverse myelitis on sagittal and axial T2-weighted images (**K,L**, respectively).

Figure 1 summarizes the typical brain and spine imaging patterns in pediatric RDS as described in the text. These are also tabulated for reference in Table 1.

**Table 1:** Imaging features of relapsing demyelinating syndromes.

**Features**	**MS**	**NMOSD**	**MOG-AD**	**ADEM**
Brain	•Discrete ovoid lesions •Size: 3 mm–2 cm •Location: Supratentorial lesions are typically periventricular (perpendicular to ventricles), juxtacortical, and cortical in location. Infratentorial lesions typically involve the brainstem, cerebellar peduncle and deep white matter paramedian medulla, peripheral location in pons, and trigeminal root entry zone •Enhancement: Typically 4 weeks but may last anywhere between 2–8 weeks. •T1 hypointensity is common and an important criterion to distinguish from monophasic illness •Course: Variable - may remain stable, enlarge or resolve. •Advances: Central vein sign, subpial demyelination and smoldering lesions	•Typical periventricular locations in periaqueductal, area postrema (often contiguous with cord), hypothalamus, thalamus. •Periventricular lesions surrounding lateral ventricles paralleling the ependymal surface unlike MS •Corpus callosum lesions paralleling long axis •Large confluent hemispheric white matter lesions •Longitudinally extensive lesions along corticospinal tracts •Non-specific white matter lesions are common •Usually no enhancement can show cloud like patchy enhancement in up to 56% •Course: Cystic changes and corresponding higher disability is common	•Multifocal deep white matter lesions with hazy boundaries •Tumefactive, poorly demarcated lesions •Cortical gray/juxtacortical white matter •Pons cerebellum, midbrain, medulla corpus callosum-focal, discrete and nodular without a specific orientation around the ventricles. A leukodystrophy-like pattern may be present. •Nodular, incomplete ring and leptomeningeal enhancement •Normal MRI despite symptoms •Non-enhancing scattered and punctate •Course is favorable in most cases with significant resolution	•Multifocal large hazy whitematter lesions •Deep gray and cortical involvement •Variable enhancement •Atypical features with MS like lesions and T1 hypointense lesions are also described •Course is less favorable than MOG positive cohort with 50% showing significant residual changes
Spinal cord	•Discrete, multiple •Cigar shaped on sagittal with short craniocaudal length (Usually <2 vertebral heights) •>3 mm •Peripheral and wedge shaped on axial images covering less than half the circumference of cord, typically along lateral and dorsal columns •Cervical >Thoracic •T1 hypointense •Enhancement less common than brain lesions nodular > incomplete ring like	•Longitudinally Extensive Transverse Myelitis (LETM) extending craniocaudally >3 vertebral heights •Central cord involvement •>50–75% cord circumference is usually involved	•LETM >short segment myelitis •Conus involvement is common. Regional cord involvement variable in different studies. •Normalization of signal on follow up is common •Variable central and peripheral cord involvement, >50% circumference involved in 60% •Enhancement in 60%	•Cord involvement is less common than MOG positive cohort. LETM is the predominant pattern
Optic nerve	•Short length, orbital segment, unilateral	•Bilateral long segment with posterior predominance, Intracranial and Chiasmal involvement common	•Longitudinally extensive or short segment bilateral or unilateral •Anterior predominant, optic disc involvement common	•Less common than MOG positive cohort

*MS, Multiple sclerosis; NMOSD, Neuromyelitis optic spectrum disorders; MOG, Myelin oligodendrocyte glycoprotein related disorders; ADEM, Acute demyelinating encephalomyelitis; LETM, Longitudinally extensive transverse myelitis*.

The key radiological patterns that emerge in the spectrum of pediatric relapsing demyelinating syndromes are listed below. An understanding of these patterns will help one approach the imaging mimics in a structured fashion.

### Optic Neuritis (ON) in RDS

The specific imaging patterns of ON in MS, AQP4-NMOSD, and MOG-AD have been described in the relevant sections. ON may be the first presentation of a systemic RDS in up to 23% of children (25) and may occur in isolation as a monophasic event (such as seen in acute disseminated encephalomyelitis—optic neuritis), recurrent event (chronic relapsing inflammatory optic neuritis) or in association with systemic RDS. On follow-up, up to 36% of children presenting with ON are eventually diagnosed with MS (26).

**Table:**
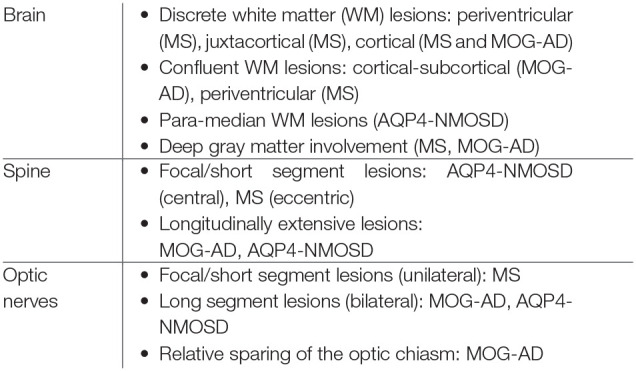

In addition to the previously described RDS, there is a wide differential diagnoses for ON in the pediatric age group. Systemic inflammatory and rheumatological disorders, vasculitis and other granulomatous disorders including sarcoidosis also need consideration and exclusion. Whilst MRI is not strictly necessary for confirmation of the diagnosis of ON, it can be helpful for assessing the pattern of optic nerve involvement and in cases where there are atypical clinical features such as insidious symptom onset, severe optic nerve pallor or acute visual loss (11).

In addition to conventional MRI, there are clinical and further imaging modalities such as spectral domain optical coherence tomography (S-OCT) that can help differentiate between the possible underlying etiology of ON with a high degree of specificity. In a recent Italian cohort study of 22 pediatric patients with ON, MOG antibody positivity was strongly associated with optic disc swelling, increased retinal nerve fiber layer (RNFL) thickness on S-OCT and better recovery (24).

## What are the Neuroradiological Mimics of the Relapsing Demyelinating Syndromes?

Prior to a more detailed discussion on disorders that may mimic pediatric demyelinating disease, it is important to note that there are several important clinical red flags that should raise concern for a mimic prior to performing any imaging (Table 2). Specifically, a relevant family history, history of drug use, fever at the onset of symptoms, multi-system involvement, or sudden onset of severe symptoms raise the suspicion of alternative pathologies. Additionally, clinical signs like deafness, psychosis, cranial neuropathy and presence of cutaneous manifestations should also prompt consideration of a mimic.

**Table 2:** Clinical red flags in the diagnosis of demyelinating disorders.

**Systemic features** Persistent fever Weight loss Anemia, nutritional deficiencies Sicca symptoms (dry eyes, dry mouth) Neuro cutaneous markers Slivery hair Alopecia, rash, conjunctivitis Rash, joint pain, hair loss, oral ulcers Paranasal sinus involvement Lung involvement Heart: Cardiomyopathy, conduction blocks Heart : Congenital heart disease Gastrointestinal symptoms Renal involvement Genital ulcers Recurrent miscarriages Bone lesions Tendon xanthomas Antecedent trauma Immuno-compromised status Known diagnosis of malignancy Multisystem involvement Drugs, toxins, chemotherapy Positive family history	Infection, autoimmune disorders Infections, autoimmune disorders, secondary neoplasms Vitamin B12 deficiency, copper deficiency Sjogren's syndrome Hypomelanosis of Ito, Sturge Weber syndrome, pigmentary mosaicisms Griscelli syndrome Biotinidase deficiency Systemic lupus erythematosus Granulomatosis with polyangiitis Granulomatosis with polyangiitis, sarcoidosis Mitochondrial disorders, sarcoidosis, infarcts Infarcts, cerebral abscess MNGIE, nutritional deficiencies secondary to malabsorption, Whipple disease, porphyria, celiac disease Mitochondrial disorders, SLE, fabry disease, systemic vasculitis Behcet's disease SLE, anti-phospholipid antibody syndrome Erdheim chester disease, histiocytosis Cerebrotendinous xanthomatosis Arterial dissections with stroke, neurological decompensation in leukodystrophies like Vanishing white matter disease, mitochondrial disorders Parasitic and fungal infections, Human Immunodeficiency virus, Progressive multifocal leukoencephalopathy, malignancies, lymphoma Secondary tumors in brain, infiltration in hematological malignancies, paraneoplastic syndromes Mitochondrial disorders Toxic leukoencephalopathy Leukodystrophies, HSP, SCA
**Optic nerve** Sudden onset visual loss Insidious onset and chronic progressive visual decline Persistent complete loss of vision Absence of RAPD Severe eye pain Uveitis Exophthalmos	CRAO, CRVO, vitreous hemorrhage, retinal detachment, acute angle closure glaucoma, cardiac emboli Toxic, nutritional deficiency, retinitis pigmentosa, open angle glaucoma, mitochondrial disorders CRAO, CRVO, vitreous hemorrhage, retinal detachment Retinitis, retinal detachment, vitreous hemorrhage, LHON Uveitis, acute angle closure glaucoma, infiltrative disorders Autoimmune disorders, infections Mass lesions, thyroid ophthalmopathy, orbital pseudotumour
**Brain** Insidious onset and steadily progressive focal symptoms Slowly progressive course with generalized involvement Stroke/stroke like symptoms Status epilepticus Dystonia, parkinsonism Early cognitive decline, dysarthria Cranial neuropathy Bilateral non-fatigable ptosis, total ophthalmoplegia Somnolence, diabetes insipidus Psychosis Meningeal signs Headache Deafness Polyradiculopathy, peripheral neuropathy Amyotrophy	Neoplasms Leukodystrophies, HSP CNS angitis, mitochondrial disorders (MELAS, pol Y), congenital disorders of glycosylation, transient ischemic attacks, CADASIL, fabry disease, migraine, seizures, cardiac emboli, moya moya disease, cerebral hemorrhage Meningoencephalitis, autoimmune encephalitis, mitochondrial disorders (pol Y), CNS angitis Anti NMDAR encephalitis, infectious encephalitis, Wilson disease Neurodegenerative disorders like MSA Lyme disease, sarcoidosis Mitochondrial disorders Sarcoidosis, lyme disease, chronic meningitis Anti NMDAR encephalitis, SLE, CNS angitis, Huntington's disease, Wilson disease Meningoencephalitis, SLE, CNS angitis, sarcoidosis Hemiplegic migraine, CNS angitis, mitochondrial disorders, SLE, sarcoidosis, meningoencephalitis, cerebral venous sinus thrombosis, Susac syndrome Mitochondrial disorders, Susac syndrome SLE, lyme disease, B12 deficiency, leukodystrophies, HMSN, Guillain Barre syndrome HMSN, lyme disease, ALS, syringomyelia, mitochondrial disorders
**Spine** Hyper-acute onset of symptoms over minutes Insidious onset and gradually progressive myelopathy Recurrent symptoms occurring at the same level Complete transverse myelitis Severe back pain	Infarct, hemorrhage HTLV myelopathy, HSP, AMN, vitamin B12 deficiency, copper deficiency Vascular malformations Infarct, trauma, bleeds, compressive lesions Vascular malformation, epidural abscess, bleeds, intervertebral disc compression

*AMN, Adrenomyeloneuropathy; ALS, Amyotrophic lateral sclerosis; CADASIL, Cerebral autosomal dominant arteriopathy with subcortical infarcts and leukoencephalopathy; CRAO, Central retinal artery occlusion; CRVO, Central retinal vein occlusion; HMSN, Hereditary motor sensory neuropathy; HSP, Hereditary spastic paraparesis; HTLV, Human T-cell lymphotropic virus; LHON, Leber hereditary optic neuropathy; MELAS, Mitochondrial encephalopathy lactic acidosis stroke like episodes; MNGIE, Mitochondrial neurogastrointestinal encephalomyopathy; MSA, Multi system atrophy; SCA, Spinocerebellar atrophy; SLE, Systemic lupus erythematosus*.

### Intracranial Mimics

**Figure 2:**
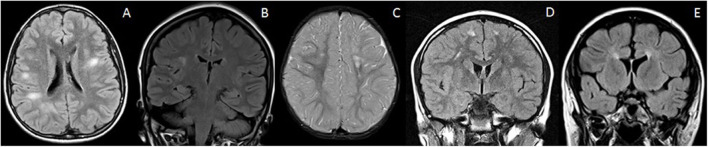
Mimics of RDS with discrete brain lesions. All these lesions on first glance could mimic RDS on the basis of imaging alone. However, the lesions do not strictly satisfy the McDonald criteria. Clinical history and follow up is therefore also vital in reaching a correct diagnosis. **(A)** NOTCH3 mutation: Axial T2-FLAIR imaging demonstrates small discrete lesions in the white matter of both cerebral hemispheres. **(B)** Leber's hereditary optic neuropathy: Coronal T2-FLAIR imaging reveals a discrete brain lesion in the right hemispheric white matter. The optic nerves were also atrophic (not shown). **(C)** Incontinentia pigmenti: Axial T2-weighted images demonstrate white matter lesions in the centrum semiovale bilaterally. There is also volume loss within the left frontal lobe. **(D)** Migraine: Coronal T2-FLAIR imaging demonstrates small discrete lesions in the frontal white matter bilaterally. These were stable on follow up and there were no clinical features of demyelination. **(E)** Hereditary spastic paraparesis: Coronal T2-FLAIR demonstrates thinning of the anterior corpus callosum and periventricular signal abnormality—“ears of the lynx” sign. There were no juxtacortical lesions.

Discrete white matter lesions of the brain can be seen as incidental or “non-specific” findings in many conditions and are, at times, erroneously reported as “possible inflammatory demyelinating” lesions (Figure 2). Increased prevalence of such “MS-like lesions” has been described in association with a diverse list of entities, including migraine, vasculitis, infections/para-infectious conditions, sarcoidosis, certain leukodystrophies, and even hemophagocytic lymphohistiocytosis (HLH) (27).

On closer inspection, however, the morphology and location of these lesions most often do not satisfy the McDonald criteria of being “periventricular” or “juxtacortical,” and such lesions are typically deep and subcortical in location. Often, a rim of normal-appearing white matter separates these lesions from the ventricular margin and cortex, respectively.

A typical example of such a mimic with discrete white matter lesions is illustrated in Case Vignette 1 of a child presenting with bilateral hemifacial spasms due to proline-rich transmembrane protein-2 (*PRRT-2*) gene mutation. *PRRT-2* gene mutations result in a truncated defective proline-rich transmembrane protein-2 in presynaptic terminals leading to an impaired neurotransmitter release. Presentation is in the form of distinct clinical syndromes which can vary with age, can overlap, and even evolve into other defined syndromes. These include benign familial infantile epilepsy (BFIE), paroxysmal kinesigenic dyskinesia (PKD), and PKD/BFIE overlap syndromes, namely infantile convulsions with choreoathetosis (ICCA) and hemiplegic migraine (HM). Scattered white matter hyperintensities may be present in imaging and can be mistaken for demyelinating lesions (28).

**Case Vignette 1—PRRT2 mutation**

A young patient presented with a history of bilateral hemifacial spasms. There were no demonstrable neurological deficits on clinical examination.

Her MRI showed multiple scattered white matter hyperintensities bilaterally. Note the rim of normal-appearing white matter separating the lesions from both the ventricular surface and cortex. Thus, her lesions did not satisfy the McDonald criteria for MS.

Because of significant clinical symptoms, genetic testing was undertaken and revealed pathogenic variations in the *PRRT-2* gene, which is a leading cause for a spectrum of paroxysmal diseases. This case illustrates how appropriate image interpretation prevents misdiagnosis even with overlapping or non-specific clinical phenotypes.

**Figure:**
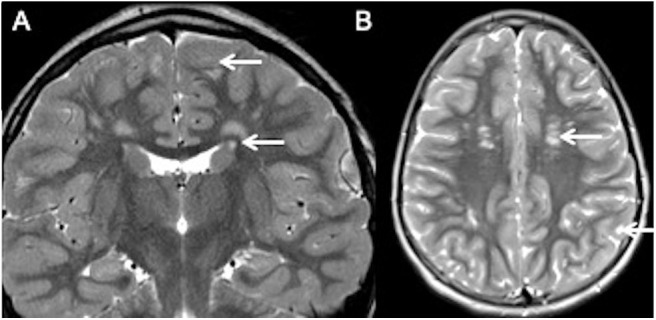

T2 coronal **(A)** and T2 axial **(B)** images show hyperintense lesions in deep and subcortical whitematter (white arrows in **B**). Note the presence of normal white matter between the lesions and ventricular surface and cortex (white arrows in **A**).

**Figure 3:**
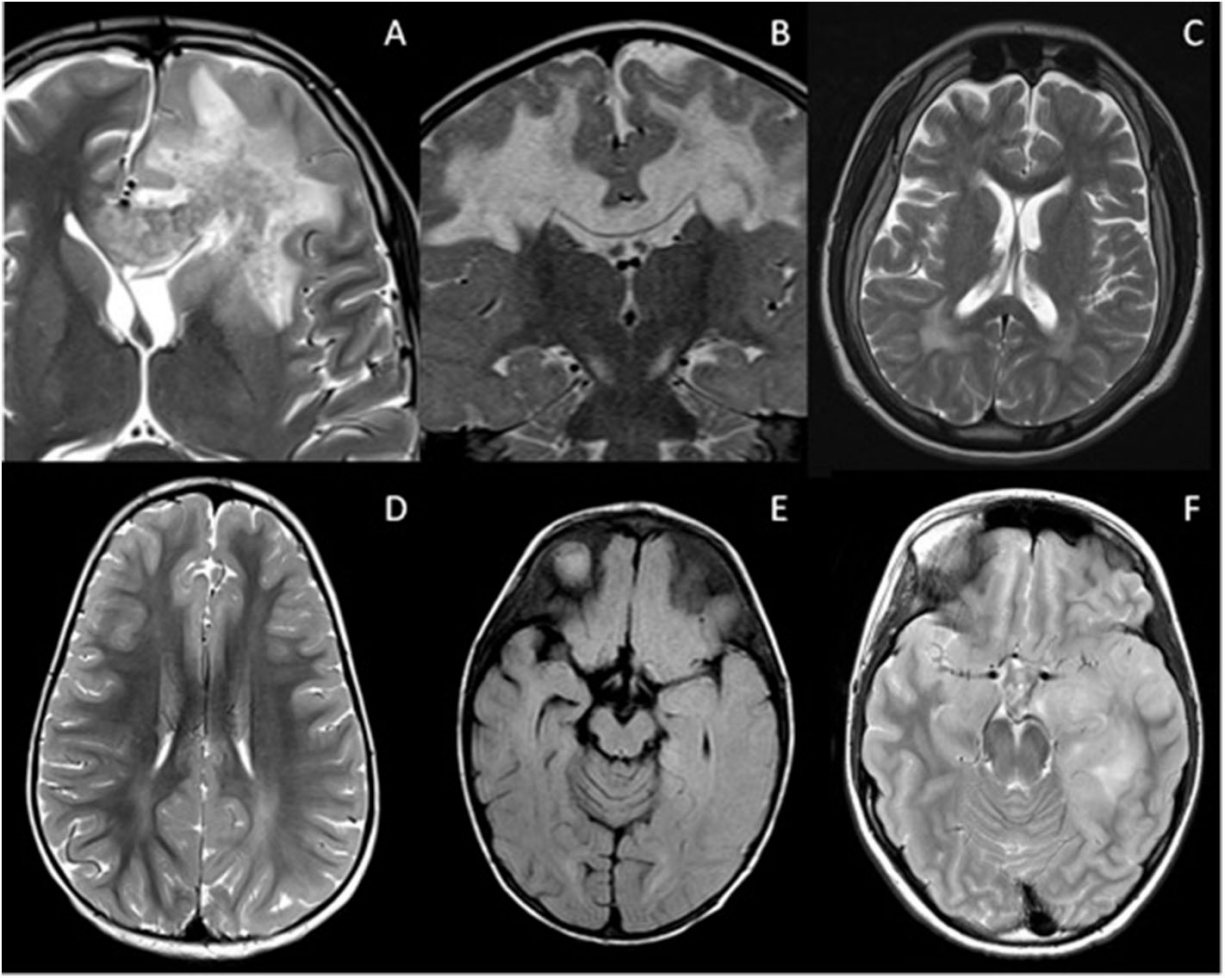
Mimics of RDS with confluent brain lesions. **(A)** Acute haemorrhagic leukoencephalopathy: Confluent white matter lesion in the left frontal lobe with intralesional hemorrhage extending into the corpus callosum. There was patchy enhancement of this lesion (not shown). Salmonella infection was confirmed on serology. **(B)** Complex 1 deficiency mitochondriopathy: Confluent white matter lesions of both cerebral hemispheres which demonstrated cavitation and restricted diffusion (not shown). **(C)** Giant axonal neuropathy due to exon 1 deletion of GAN gene: Axial T2-weighted image illustrates signal abnormality within the frontoparietal white matter and parenchymal volume loss. **(D)** Subacute sclerosing panencephalitis: White matter signal abnormality is noted within the parietal lobes bilaterally, but is more extensive in the left cerebral hemisphere where there is blurring of the gray-white matter margin. **(E)** Anti NMDA receptor antibody encephalitis: Axial FLAIR imaging demonstrates signal abnormality within the caudate nuclei bilaterally, the right putamen and left globus pallidus. There is also involvement of the right sided subinsular white matter and posterior limb of the internal capsule. The appearances could be mistaken for ADEM. **(F)** Glioma: Axial T2-weighted image demonstrates white matter signal change in the left temporal lobe with swelling of the cortex. This was followed up, and subsequently biopsied due to growth and enhancement.

Intracranial lesions can also be confluent in a variety of disease states, mimicking primary or secondary progressive MS when in the posterior periventricular regions, or MOG-AD when more cortical-subcortical in location (Figure 3). These include the encephalitides, leukodystrophies, and even periventricular leukomalacia (PVL) in the context of white matter injury of prematurity.

When these lesions occur infratentorially, such as in the case of rhombencephalitis or Alexander disease, they may be confused for AQP4-NMOSD, particularly if there is involvement of the area postrema, as shown in Case Vignette 2.

**Case Vignette 2—Juvenile Alexander disease**

A teenager presented with severe vomiting. His premorbid health was normal except for mild intellectual disability.

His MRI imaging showed focal hyperintensity and swelling of the area postrema with intense enhancement. CSF studies showed no oligoclonal bands. Both AQP4 and MOG antibodies were negative in serum and CSF. He was subsequently diagnosed with juvenile Alexander disease.

This case illustrates that many diseases can have common areas of selective vulnerability. *Homogenous intense enhancement and absence of AQP4 antibodies* were the features that led to further investigation and alternate diagnosis.

**Figure:**
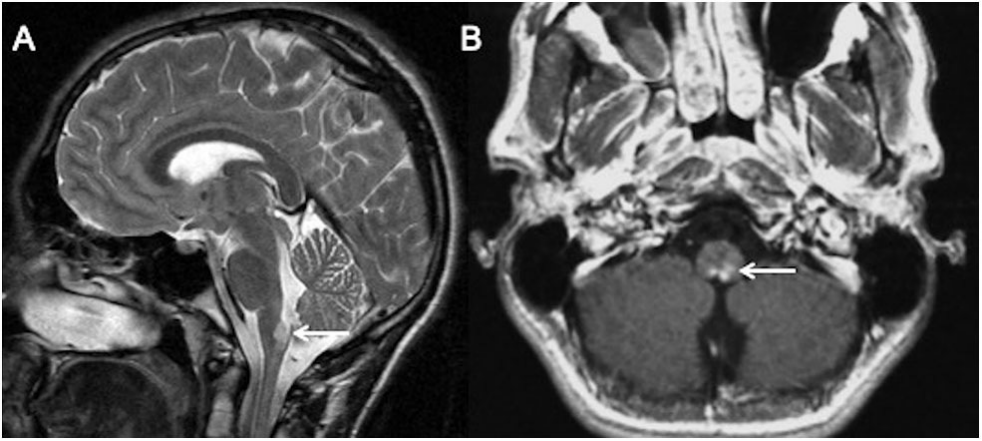

T2 sagittal **(A)** and T1 post contrast axial **(B)** images show hyperintensity and swelling of area postrema with intense nearly homogenous enhancement (white arrows).

Alexander disease (AD) is a glial fibrillary acidic protein (GFAP) related astrocytopathy characterized by an abundance of Rosenthal fibers in astrocytes, particularly in subpial and subependymal locations. The distribution of lesion in AD is reminiscent of AQP-4 NMOSD (29). Juvenile and adult forms of AD preferentially involve the brainstem and cerebellum. Periventricular, periependymal, midbrain, and brainstem lesions often associated with patchy areas of enhancement can be misinterpreted as AQP4-NMOSD (29).

### Infections and Para-infectious Disorders

Infections account for a large group of potential MS mimics. Isolated, multifocal, or confluent lesions of the white and gray matter are often seen in infections, presenting in a rather non-specific manner (30). Clinical and laboratory findings play an important role in distinguishing infectious/para-infectious diseases from demyelinating disorders (31, 32).

Imaging red flags concerning for infectious mimics of RDS include meningeal enhancement (meningitis), complete ring enhancement with restricted diffusion (abscess), venous sinus thrombosis, calcification as in neurocysticercosis and toxoplasmosis, and bilateral striatal and thalamic involvement as commonly in viral encephalitis. Acute haemorrhagic leukoencephalitis (AHLE) is thought to be another post-infectious phenomenon presenting with white matter demyelination. It can occur after viral or bacterial infections.

Borrelia burgdorferi causing Lyme disease deserves special mention as the CNS imaging demonstrates “MS-like” subcortical and periventricular white matter lesions, including the callososeptal interface (32). The presence of cranial and spinal nerve enhancement, as well as meningeal enhancement, are important distinguishing features of Lyme disease on neuroimaging.

Epstein Barr virus (EBV) encephalitis also presents with multiple lesions in the cerebral cortex/subcortical white matter, thalami, basal ganglia, and, sometimes, brainstem or cerebellum. Rarely, it can cause optic neuritis, further confounding the diagnosis (33).

Cytomegalovirus (CMV) has a predilection for the ependymal, germinal matrix, and capillary endothelial cells. The pattern of involvement may mimic MS with a periventricular distribution of lesions (30).

Multifocal lesions, usually related to a microvascular etiology can also be observed in viral diseases such as HTLV-1 and HIV (34). The lack of contrast enhancement distinguishes them from demyelination. Calcification of the basal ganglia or frontal white matter is also a useful discriminator of HIV (35, 36).

Progressive multifocal leukoencephalopathy (PML) is an opportunistic infection caused by the JC polyomavirus (JCV). Supratentorial white matter lesions are typically multifocal, asymmetric, bilateral, and at times with confluent lobar involvement. PML can affect the deep gray nuclei also involve the brainstem and cerebellum. Generally, there is no enhancement or mass effect (34).

Acute disseminated encephalomyelitis (ADEM), an autoimmune-mediated white matter disorder that often follows a viral upper respiratory tract infection (EBV, influenza A, coronavirus), can appear very similar to MS. It is characterized by multifocal lesions of the deep and juxtacortical white matter, sometimes involving the cortex, as well as thalami, basal ganglia and also the brainstem and cerebellum. A history of recent upper respiratory infection or vaccination is often present and should be actively sought.

“Open ring” or incomplete peripheral enhancement deemed specific for demyelinating lesions, particularly MS, is useful for differentiating between demyelination and other space-occupying lesions like neoplasm or an abscess. However, CNS infections such as neurocysticercosis and occasionally tuberculosis, as well as ADEM can also be associated with MS-like “open ring” enhancement (32).

### Subacute Sclerosing Panencephalitis (SSPE)

SSPE is a progressive measles virus mediated encephalitis that may present with brain MRI findings similar to a demyelinating disease. It is believed to be associated with an immature immune system and is seen in children with the onset of the primary infection in the first two years of life (37). On imaging, multifocal, bilateral but asymmetric lesions of the cortex and subcortical white matter are seen. As the disease progresses, there is usually involvement of the parietal and temporal lobes and the lesions extend into the periventricular white matter and corpus callosum. Mass effect and contrast enhancement may be present during this phase (38, 39).

While there may be some overlap on imaging between SSPE and demyelination, the clinical examination is very specific, characterized by insidious onset of behavioral changes followed by mental deterioration. Seizures, myoclonus, dementia, and inexorable progression to death occur.

Pathological findings include predominant involvement of the gray matter with white matter demyelination, perivascular lymphocytic cuffing, intracellular viral inclusions, neuronophagia, and gliosis (37).

### Posterior Reversible Encephalopathy Syndrome (PRES)

PRES may rarely be confused as demyelination mimic on imaging, especially when the lesions are discretely distributed in the white matter, or when there is considerable cortical-subcortical involvement. With PRES however the clinical context is extremely relevant. Usually, there is an apparent predisposing factor such as chemotherapy, hypertension or an underlying systemic condition. The clinical presentation may however partly overlap with RDS, particularly MOG-AD with features such as encephalopathy and seizures (40).

The exact pathophysiology of PRES remains unclear however it is hypothesized to relate to cerebral vascular auto-regulatory and endothelial dysfunction. PRES itself may be considered a misnomer as the lesions are not always located posteriorly, nor are they always reversible (41). The topographical patterns of PRES in children differ slightly from adults, with frontal lesions being more common than the parieto-occipital pattern, the dominant pattern in adults (42–44). Increased incidence of cerebellar involvement and contrast enhancement has also been noted (42) however this has been disputed by others (43, 45). In addition, involvement of the gray-matter structures, corpus callosum, and brainstem has also been described. Hemorrhage, enhancement and abnormality on diffusion weighted imaging is a less common feature (46).

### Genetic Leukodystrophies

Leukodystrophies can share similarities with demyelinating disorders on imaging. In addition, demyelinating disorders have been shown to co-exist in patients with mitochondriopathies. It remains unclear whether the mutations underpin an autoimmune trigger for demyelination or if these cases are indeed unusual presentations of mitochondrial disorders (47). Clinical indicators of a mitochondrial etiology include the presence of ataxia and myopathy, external ophthalmoplegia, refractory optic neuropathy/neuritis, seizures, pigmentary retinopathy, peripheral neuropathy, or cardiomyopathy/cardiac conduction defects.

Leber's hereditary optic neuropathy (LHON), a mtDNA mutation disorder with specific point mutations in complex 1, occurs in patients with MS at a frequency ~50 times greater than in the general population (48). Another mitochondrial disorder with progressive optic atrophy is optic atrophy type 1. MS-like white matter hyperintensities involving the brain and cord have been described as a feature in both these disorders, although enhancement has never been described (49).

*POLG* includes a set of nuclear genes with the function of maintaining the mtDNA pool through mtDNA duplication. POLG related disorders have vastly overlapping clinical phenotypes of varying severity. Unusually, a relapsing-remitting illness with MS-like lesions and ADEM like white matter lesions has been described (47, 50, 51).

Certain leukodystrophies manifest as small vessel disease and therefore can mimic inflammatory demyelination. CADASIL (NOTCH3), CARASIL (HTRA1), 6p25 deletion syndrome, cerebral small-vessel diseases (FOXC1 and PITX2) are the typical entities within this group. Whilst most of these disorders have an onset after the 3rd decade, pediatric-onset disease has been rarely described.

*NOTCH3* encodes a transmembrane protein expressed in vascular smooth muscles and heterozygous mutations leading to cerebral autosomal dominant arteriopathy with subcortical infarcts and leukoencephalopathy (CADASIL). The clinical features include recurrent subcortical ischaemic strokes with cognitive decline. Patchy multifocal white matter abnormalities involving the deep and periventricular white matter are common in the described pediatric cases (52–54).

Fabry's disease, a lysosomal disorder with large and small vessel microangiopathy, is another MS-mimic with many patients described as previously wrongly labeled as definite-MS based on revised McDonald criteria (55).

### Vasculitis

CNS vasculitis can be classified into primary angiitis and secondary vasculitis. Primary angiitis of the CNS (PACNS) is inflammation limited to the arteries of the CNS (56). Secondary CNS vasculitis is associated with multiple etiologies, such as systemic infectious or inflammatory disease, collagen vascular diseases, malignancy, drugs, and substance abuse.

Imaging, although variable and sometimes transient, shows multiple small/punctate lesions or even tumefactive enhancing lesions in the subcortical white matter and gray matter, more often affecting the anterior than posterior circulation. Basal ganglia involvement is frequently noted. Diffuse leptomeningeal enhancement may also be seen (57).

Additional findings include microhemorrhages and multifocal infarction (58). Systemic involvement may help make the diagnosis but a brain biopsy may be eventually needed.

### Hemophagocytic Lymphohistiocytosis (HLH)

HLH is severe systemic hyperinflammatory syndrome of a dysfunctional immune response characterized by unchecked proliferation of natural killer cells and T-lymphocytes (59). While an underlying genetic defect is responsible for the primary form, the secondary form usually follows infectious, malignant or autoimmune triggers (59). Primary and secondary HLH are further classified based on the genetic defect and the resulting disrupted immune process, and the inciting trigger (60). The familial forms can also be associated with immune deficiency syndromes (Chédiak-Higashi syndrome 1, Griscelli syndrome 2, and X-linked lymphoproliferative syndrome) in which HLH can develop sporadically during the disease course (59).

CNS involvement is common in both inherited and acquired forms of HLH. The most common imaging pattern is asymmetric confluent white matter lesions with subcortical and deep white matter distribution (61). Cerebellar and deep gray nuclear involvement is also common and these features closely mimic MOG related and antibody-negative demyelinating syndromes.

A nodular perivascular pattern of enhancement, which is often seen, may help in differentiation. Very occasionally more focal well-circumscribed lesions may be present mimicking MS lesions. Some of these cases were previously incorrectly labeled as CLIPPERS (chronic lymphocytic inflammation with pontine perivascular enhancement responsive to steroids).

Case Vignette 3 demonstrates the typical clinico-radiological picture of HLH in the setting of Griscelli syndrome Type 2, a primary HLH associated syndrome with immunodeficiency and hypopigmentation caused by dysfunction in T-cell vesicle docking due to *RAB27A* mutations (59).

**Case Vignette 3—Griscelli type 2 syndrome (HLH)** An adolescent presented to the neurology services with a long history of recurrent episodes of blurred vision and ataxia. Clinically the patient was diagnosed as demyelination.

CSF analysis showed normal protein with no cells or organisms. Genetic testing subsequently confirmed Griscelli type 2 syndrome. The suspicion was also raised on the grounds of the clinical picture which included abnormal hair pigmentation.

Although MOG-AD can also present with large fluffy ill-defined white matter lesions and show a predilection to cerebellar peduncles, the pattern of enhancement seen here is quite atypical. Nodular intense enhancement in a perivascular distribution is more characteristic of the inflammatory and vasculitis spectrum of disorders. HLH also demonstrates a pontine and cerebellar peduncle predominant distribution.

**Figure:**
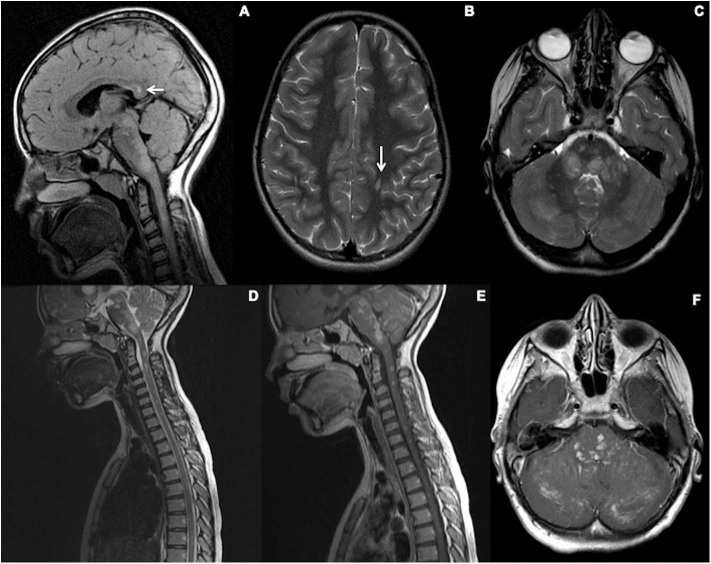

Top row **(A–C)**: Initial imaging at age 12. **(A)** Sagittal T2 FLAIR-weighted imaging through the brain demonstrates evidence of signal hyperintensity within the calloso-septal interface, callosal splenium (white arrow), dorsal brainstem, and cervical spine. **(B,C)** Axial T2-weighted sequences of the brain show a juxtacortical lesion in the left perirolandic region (arrow) and ill-defined areas of abnormal signal in the pons and cerebellar hemispheres bilaterally (R>L).

Bottom row **(D–F)**—Initial imaging at age 12. **(A,B)** Sagittal T2 and post-contrast T1-weighted sequences show the extent of signal abnormality within the brainstem and spinal cord. All the T2 hyperintense parenchymal lesions show enhancement. F-Axial post-contrast T1 Weighted sequence through the posterior fossa shows enhancement corresponding to the T2 hyperintense areas of abnormal signal in the pons and cerebellar hemispheres bilaterally (R>L). In addition, there is folial enhancement suggesting pial involvement.

### Anti N-Methyl D-Aspartate Receptor Encephalitis (Anti-NMDARE)

Anti-NMDARE presents with a characteristic clinical spectrum of abnormal behavior, speech dysfunction, memory/cognitive disturbance, seizures, movement disorder, and even decreased level of consciousness and autonomic dysfunction (62). CSF may show pleocytosis and presence of oligoclonal bands (63).

Knowing that there is an overlap between anti-NMDARE and demyelinating disease (AQP4-NMOSD and MOG-AD) is important as patients may present clinically with concurrent or separate episodes of demyelination and/or atypical psychomotor features. The presence of different antibodies has implications for treatment and prognosis. Testing for anti-NMDA, AQP4 and MOG antibodies may therefore be warranted in such cases (64).

There is a higher prevalence of anti-NMDARE in children with herpes simplex virus (HSV) 1 IgG antibodies, including those without clinically evident encephalitis (65). Although less common in children, there is a strong association of ovarian teratomas in young women (46–70%) with anti-NMDARE (62, 66).

MR imaging is often normal at initial presentation, but when abnormal shows non-specific cortical and subcortical lesions with no clear localization. Optic neuritis can also be a feature (62). Striatal necrosis, hippocampal, or global atrophy is present in progressive stages (67).

### Neurosarcoidosis (NS)

Neurosarcoidosis is a disorder of unknown etiology, characterized by non-caseating granulomas histologically. It is rare in the pediatric population. NS can affect any part of the nervous system. Uveitis, optic neuropathy, hypothalamic dysfunction, mass-like brain lesions, and encephalopathy are features seen in pediatric NS (68). The most common neurological complication of sarcoidosis is cranial neuropathy, with a distinct predilection for cranial nerves II, III, and VII. Facial nerve palsy may be bilateral. Optic neuritis, often bilateral, has been observed as an initial disease presentation in up to 35% of cases (69).

Imaging in children with neurosarcoidosis more commonly shows enhancing parenchymal lesions than its adult counterpart. Discrete to confluent white matter and cerebellar hyperintensities with punctate or discrete enhancing lesions are noted. Leptomeningeal, pituitary stalk or cranial nerve enhancement are additional features (70, 71).

### Rare Disorders With White Matter Lesions

Neurocutaneous and microangiopathic disorders with asymmetrical CNS white matter involvement can also mimic pediatric demyelinating disorders on imaging and should be borne in mind.

### Incontinentia Pigmenti (IP)

IP, an X-linked dominant disorder, is caused by mutations in nuclear factor (NF)-*k*-B *e*ssential m*o*dulator (NEMO) gene (72). Clinically, affected neonates present with inflammatory skin abnormalities, encephalopathy, and seizures (73). Imaging in neonates shows asymmetrical lobar or hemispheric cortical and white matter oedema with diffusion restriction, often labeled as an encephalitis. On follow-up, atrophy, scattered white matter hyperintensities, cortical laminar necrosis and ex-vacuo ventriculomegaly are usually present and can be mistaken as seqeulae of PVL (72, 74).

### Hypomelanosis of Ito

Hypomelanosis of Ito, a disorder of chromosomal mosaicism, with several underlying genetic defects has typical hypopigmented skin lesions along the lines of Blaschko. White matter involvement in the form of asymmetrical deep and periventricular white matter hyperintensities can be present, often with dilated cystic or perivascular spaces (74).

### Hereditary Spastic Paraparesis (HSP)

Whilst periventricular hyperintensities may be present on imaging in HSP, the clinical phenotype of progressive spastic paraparesis with a relevant family history serve as useful differentiators (75). Additional neuroimaging clues may also be present, such as thinning of the corpus callosum.

### Susac Syndrome (SS)

SS is another rare condition in the pediatric age group. It is an autoimmune microangiopathic disorder resulting in occlusion of the branch retinal arteries and microinfarction of the central nervous system and cochlea. The onset of all three characteristic features at presentation is seen in only a minority of patients, reported as low as 13% (76, 77). Although primarily a disease affecting young women between the age of 20 and 40 years, SS has been reported in patients aged 7–70 years (78). The characteristic finding on MRI is the involvement of the middle layers of the corpus callosum with T2 hyperintense punched out lesions referred to as “snowball” lesions. Acute lesions demonstrate punctate enhancement. Leptomeningeal enhancement occurs in 30% of patients (79).

## Optic Nerve Mimics

**Figure 4:**
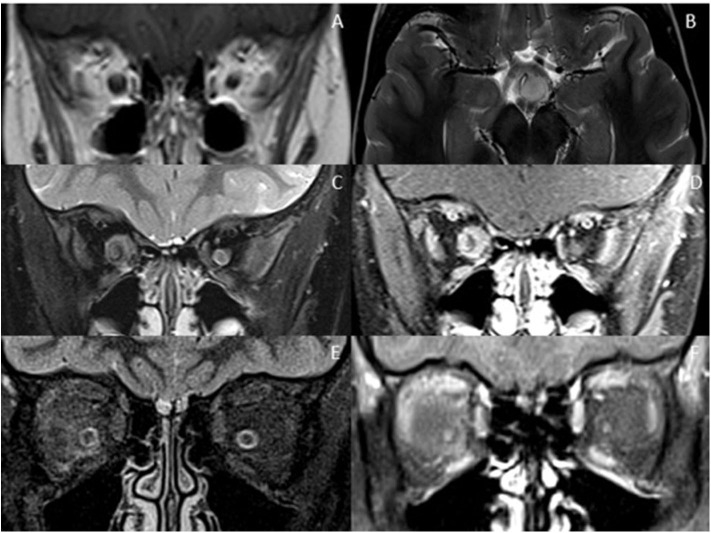
Optic nerve mimics of RDS. **(A,B)** Optic pathway glioma: The coronal T1-weighted image with contrast shows an expanded intra-orbital right optic nerve. The optic chiasm is expanded on T2-weighted axial imaging **(B)**. **(C,D)** Optic nerve sheath meningioma: On coronal STIR **(C)** and coronal T1-weighted image with contrast **(D)** there is thickening and enhancement of the right optic nerve sheath complex. **(E,F)** Orbital sarcoidosis: On coronal STIR **(E)** and coronal T1-weighted image with contrast **(F)** there is stranding of the intraconal fat, minimal perineural thickening and enhancement.

Optic nerve involvement with swelling, T2- hyperintensity and enhancement is not specific for demyelinating disorders and can be seen in other inflammatory, infective, ischemic, toxic, and neoplastic conditions (Figure 4). That said, discrete brain lesions, as seen in MS, are not demonstrated in many of these mimics. Given the non-specific nature of optic neuritis, the morphology of coexisting brain and spinal cord lesions is often the most helpful feature in diagnosis.

Extra-neural involvement of other orbital structures is also a good indicator that one is not dealing with a primary demyelinating disorder, but rather a granulomatous, infectious or neoplastic cause. In addition, abnormal dural and leptomeningeal enhancement should also raise the suspicion of granulomatous disease, particularly sarcoidosis (68, 80).

Viral infections can present with optic neuritis. Specifically, EBV and Lyme disease should be considered. In such cases, there may be additional intracranial imaging findings which should be carefully sought (81, 82).

Tumors are typically less challenging to differentiate. Optic nerve glioma can be distinguished by expansion, relatively lessT2 hyperintensity and paucity of enhancement of the nerve, whereas optic nerve sheath meningiomas are characterized by enhancing expansion of the optic nerve sheath complex along with tram-track calcification, usually better shown on CT (83).

## Spinal CORD Mimics

**Figure 5:**
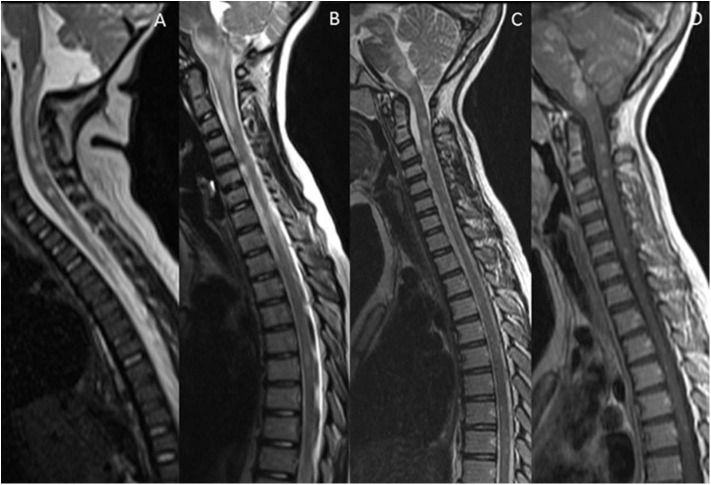
Mimics in the spine—short segment spinal lesions. **(A)** Infectious myelitis secondary to cytomegalovirus: Multifocal short segment T2 hyperintense lesions are noted in the cervical and upper thoracic cord. **(B)** Neurofibromatosis 1: Intramedullary foci of abnormal signal intensity (FASI). **(C,D)** Hemophagocytic Lymphohistiocytosis: Short segment enhancing lesions. These have a more punctate morphology.

**Figure 6:**
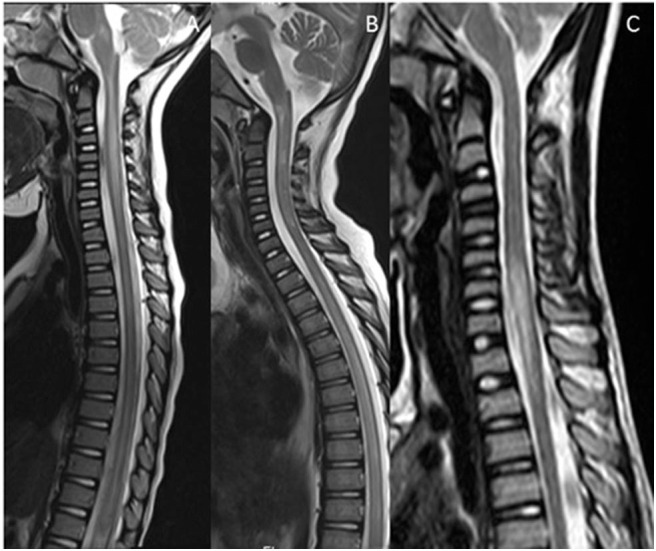
Mimics in the spine—longitudinally extensive spinal lesions. **(A)** Rhombencephalomyelitis: This was confirmed as mycoplasma on serological testing. **(B)** Biotinidase deficiency: Confirmed on genetic and enzymatic testing in a child with skin rash, seizures and alopecia. The appearances are similar to those seen on the RDS spectrum LETM however the clinical features are not typical of RDS. **(C)** Fibrocartilaginous embolism: Note the signal abnormality in the cord as a result of infarction. There was restricted diffusion within the lesion (not shown). The likely source was the C3/4 intervertebral disc which demonstrates a reduction in signal (white arrow).

For practical purposes, the spinal mimics of relapsing demyelinating disorders can be subdivided into diseases with short segment cord involvement (Figure 5), and those with a longitudinally extensive involvement (LETM) (Figure 6).

## Short Segment Spinal CORD Involvement

### Foci of Abnormal Signal Intensity (FASI) in Neurofibromatosis Type 1 (NF1)

FASI's have been described within the spinal cord of children with NF1. Short segment, non-enhancing intramedullary lesions demonstrating stability or regression on follow-up have been described (84). These are almost never found in isolation and the classic brain and orbit findings will help make the diagnosis.

### Vasculitis

CNS vasculitis with spinal involvement may occur as a part of a systemic vasculitic process such as Bechet disease, systemic lupus erythematosus or granulomatosis with polyangiitis. Primacy CNS vasculitis involving the spinal cord only is a rare entity (85). The imaging features of spinal vasculitis are non-specific and include intrinsic T2 hyperintense lesions which may or may not enhance after contrast (86).

### Intramedullary Tumors

Intramedullary spinal tumors represent 4–10% of all central nervous system tumors. They are predominantly of glial origin and account for up to 35% of all intradural tumors in children (87). The imaging features of intramedullary spinal tumors can overlap with inflammatory conditions. Location wise, intrinsic cord tumors can be located centrally or eccentrically within the cord, as typically in the case of ependymomas and astrocytomas, respectively. T2 signal hyperintensity may be present and cord expansion can be a variable feature. They may be short or long in terms of segmental involvement. The tumors may enhance, be associated with hemorrhage, tumoral cysts, and syringohydromyelia (88). Mass effect and enhancement, when present can sometimes be helpful in distinguishing from a demyelinating process.

## Longitudinally Extensive CORD Involvement

Longitudinally extensive transverse myelitis (LETM) presents clinically with a bilateral, symmetric or asymmetric sensorimotor and autonomic spinal cord dysfunction. Typically, there is a clearly defined sensory level and a progression to the nadir of clinical deficits between 4 h and 21 days after symptom onset. The primary mimics of spinal demyelination with a longitudinally extensive pattern include tumors (covered above), sarcoidosis, infections, vascular abnormalities, nutritional deficiencies such as vitamin B12 or copper deficiency, and rarely, certain metabolic entities such as biotinidase deficiency, and mitochondriopathies.

### Sarcoidosis

Spinal disease may occur in the absence of intracranial disease. Myelopathy associated with sarcoidosis is typically in the form of longitudinally extensive spinal cord lesions affecting the dorsal part of the cord, extending laterally at times as a crescent, but also less commonly the anterior aspect of the cord (89). Occasionally, central canal enhancement may be present. Long linear sub-pial enhancement, and persistence of enhancement for months despite pulsed and oral corticosteroid treatment, is highly suggestive of spinal cord sarcoidosis.

### Infectious and Para-infectious Disorders

Clinically, infective myelitis can present in a similar fashion to idiopathic myelitis with constitutional symptoms and fever. The pathogenic cause may be viral, bacterial, fungal or parasitic. Certain findings on MRI may help point toward a particular infectious pathogen.

In Lyme disease, MRI shows early enhancement of the pial region followed by non-specific T2 hyperintensities and enhancement of the cord parenchyma (33, 90).

CMV is associated with thickening, clumping, and enhancement of nerve roots and leptomeninges along the conus medullaris, often with associated long-segment T2 high signal of the cord (30).

Herpesviruses, including types 1, 2, 6, and 7 are most frequently associated with myelitis and share an overlapping imaging presentation, characterized by long-segment T2 high signal with variable enhancement (91).

In varicella-zoster myelitis, when a concomitant skin lesion is present (in 33% of patients), the dorsal root and posterior horns of the spinal cord are affected and usually correspond to the affected dermatome (92). Additionally, the MRI may show single or multiple lesions, with or without enhancement, associated with marked edema (93).

Another presentation of viral diseases, characterized by a poliomyelitis-like syndrome is seen in the picornavirus family (enterovirus 71, poliovirus, and, less commonly, coxsackievirus A and B) and in some flaviviruses, including Dengue and West Nile viruses. Imaging demonstrates unilateral or bilateral high signal on T2 sequences in the anterior horns of the spinal cord across multiple segments with variable enhancement (91).

Spinal cord presentation in HTLV-1 usually reflects involvement of the dorsolateral columns, with T2 high signal long-segment involvement of the lateral columns, less commonly extending to the dorsal columns, occasionally with enhancement (32, 91). HIV is another possible differential diagnosis for imaging abnormalities along the dorsolateral medullary column (91).

Mycoplasma is one of the most common bacterial infections resulting in post-infectious transverse myelitis. The imaging findings are not specific and a high index of suspicion is needed to exclude it as best as possible (93).

Neurocysticercosis and occasionally tuberculosis, in some stages, are associated with MS-like “open ring” enhancing lesions as mentioned previously (32).

### Vascular Abnormalities

Acute spinal cord infarction results in a sudden onset anterior spinal artery syndrome, with loss of function of the ventral two-thirds of the spinal cord, pain, and characteristic dissociative sensory disturbance. There is usually a cardiovascular risk factor for the development of cord infarction (94). On MRI, there is preferential involvement of the gray matter. The appearances may mimic LETM, though the cord lesion typically demonstrates a characteristic appearance of “owl eyes or snake eyes” on axial images, due to involvement of the gray matter of the anterior horns of the spinal cord. The presence of restricted diffusion in such cases can be helpful (94).

Fibrocartilage embolism should be considered when there is an additional finding of altered signal in the disc or in the posterior aspect of the vertebral body (95).

Spinal vascular malformations is an umbrella term encompassing a number of entities which include arterio-venous malformations (AVM), dural arterio-venous fistula (dAVF), cavernous malformations, and capillary telangiectasias. A spinal vascular malformation should be included in the differential diagnosis for any child who presents with slowly progressive or acute symptoms of radiculopathy or myelopathy.

### Nutritional Deficiencies

Nutritional deficiencies can cause appearances in the spinal cord that can mimic findings similar to those of transverse myelitis.

The characteristic clinical triad of subacute combined degeneration caused by vitamin B12 deficiency includes symmetric diminished vibration sense, pyramidal signs, and peripheral neuropathy (96, 97).

Symmetric T2 signal hyperintensity with a general lack of enhancement in the lateral and dorsal columns has been reported to be the characteristic neuroimaging finding (94). In such cases, the brain should also be imaged, as brain lesions in vitamin B12 deficiency resemble that of MS with T2 hyperintensities in the periventricular white matter.

A myelopathy similar to that seen in vitamin B12 deficiency with the involvement of the dorsal column and corticospinal tracts also may be seen in copper deficiency myelopathy (54, 98).

## Conclusion

RDS in children encompass a diverse spectrum of entities. There are a multitude of acquired and genetic disorders that can mimic RDS in children both clinically and radiologically. Furthermore, false negative test results for antibodies associated with RDS, as well as overlap with other syndromes such as anti-NMDARE can make the process of reaching an accurate diagnosis challenging.

A knowledge of the specific and distinct MRI patterns and clinical red-flags can help differentiate between the relapsing demyelinating syndrome subtypes and their clinical and radiological mimics.

## Author Contributions

PM and NR: compilation of manuscript. SC: contribution to manuscript, final manuscript review, and compilation of images. KMa: project oversee, final manuscript reviews, and corrections. SS: manuscript outlay and proof reads. KMu: neurology input and tables. All authors contributed to the article and approved the submitted version.

## Conflict of Interest

The authors declare that the research was conducted in the absence of any commercial or financial relationships that could be construed as a potential conflict of interest.
